# QTL for Stress and Disease Resistance in European Sea Bass, *Dicentrarhus labrax* L.

**DOI:** 10.3390/ani10091668

**Published:** 2020-09-16

**Authors:** Dimitrios Chatziplis, Stavroula Oikonomou, Dimitrios Loukovitis, Dimitrios Tsiokos, Athanasios Samaras, Arkadios Dimitroglou, Lefteris Kottaras, Kantham Papanna, Leonidas Papaharisis, Costas Tsigenopoulos, Michail Pavlidis

**Affiliations:** 1 Laboratory of Agrobiotechnology and Inspection of Agricultural Products, Dept of Agricultural Technology, School of Geotechnical Sciences, International Hellenic University, Alexander Campus, P.O. Box 141, 57 400 Sindos, Thessaloniki, Greece; chatz@ihu.gr (D.C.); valiaekonomou@hotmail.com (S.O.); 2 Department of Genetics, Development and Molecular Biology, Aristotle University of Thessaloniki, University Campus, 54124 Thessaloniki, Greece; 3 Research Institute of Animal Science, ELGO Demeter, 58100 Paralimni, Giannitsa, Greece; tsiokosd@rias.gr; 4 Department of Biology, University of Crete, GR-714 09 Heraklion, Greece; sam_thanasis@yahoo.gr (A.S.); pavlidis@biology.uoc.gr (M.P.); 5 Department of Research & Development, Nireus Aquaculture SA, 341 00 Chalkida, Greece; a.dimitroglou@nireus.com (A.D.); l.kottaras@nireus.com (L.K.); k.papanna@nireus.com (K.P.); l.papaharisis@nireus.com (L.P.); 6 Institute of Marine Biology, Biotechnology and Aquaculture (IMBBC), Hellenic Centre for Marine Research (HCMR), 71003 Heraklion, Crete, Greece; tsigeno@hcmr.gr

**Keywords:** *Dicentrarchus labrax*, QTL, body weight, vibriosis, biochemical markers, hormonal markers

## Abstract

**Simple Summary:**

Over the last decades, many genetic tools have been developed in order to improve our knowledge and understanding of the links between physiology and genetics. In our research, European Sea Bass was selected in order to study stress response physiological indicators in relation to growth and disease resistance. Therefore, DNA samples were collected in order to identify any potential relation between the aforementioned traits and their genetic component. Genomic areas related to body weight and stress response were detected. No genomic areas related to disease resistance were identified. Based on the results, fish that hold the genetic information for increased body weight and improved stress response could be used for genetic improvement purposes.

**Abstract:**

There is a growing interest in selective breeding in European sea bass (*Dicentrarchus labrax*), especially regarding family selection based on growth performance. In particular, quantitative trait loci (QTL) identification in sea bass enhances the application of marker-assisted breeding for the genetic improvement of the production traits. The aims of the study were to identify potential QTL affecting stress and immunological indicators, body weight, and mortality after vibriosis injection in sea bass as well as to estimate heritability and genetic/phenotypic correlations for the aforementioned traits. To this end, stress test was performed on 960 offspring and a sub-group of them (420) was selected to explore the mortality after vibrio injection. Selective genotyping was performed in 620 offspring for 35 microsatellite markers and distributed into 6 linkage groups. The length of the genetic linkage map was 283.6 cM and the mean distance between the markers was 8.1 cM. QTL affecting body weight in three different growth periods detected on linkage groups LG1, LG4, LG6, and LG14. A QTL associated with weight in early growth stages (290–306 days post-hatching) was also identified on LG3. QTL analysis confirmed the existence of QTL affecting cortisol levels, on LG3 and LG14. Moreover, new QTL affecting only cortisol and glucose levels were detected on LG1 and LG23. No QTL affecting hormonal or biochemical marks was found on LG4 and LG6. Heritability of cortisol, lysozyme levels, and mortality were high (0.36, 0.55, and 0.38, respectively).

## 1. Introduction

Quantitative genetic variation characterizes many traits of economic importance in livestock and also welfare of farmed animals. Variation in such complex traits is often influenced by a number of different quantitative trait loci (QTL), as well as environmental factors. QTL identification in commercially important species promotes the application of marker-assisted breeding for the genetic improvement of production traits. Studies with simulated and real data have shown that the utilization of marker information in breeding programs is quite beneficial in terms of time and cost efficiency by increasing the accuracy of selection and decreasing the generation interval compared to selection based only upon phenotype [1,2,3,4]. Concerning the use of genomic technologies, aquaculture breeding schemes tends to lag behind their terrestrial livestock counterparts; in Europe, it is evident that only part of the fish aquaculture industry fully exploits selective breeding to the best advantage [5]. Selective breeding is still relatively limited in few aquaculture species and the majority of breeding programs is based on family selection despite the extensive availability of molecular genetic tools for many different fish species [6,7].

Studying the stress physiology of farmed animals and understanding how they respond to stressors is of great importance in their performance and welfare. Much attention has been given during the last decades to the observation that individuals of the same species may show consistently divergent physiological and behavioral responses to a stimulus or stressor. These responses in vertebrates, according to the allostasis theory, follow two main coping styles: the proactive and the reactive [8]. These two different coping styles are thought to represent alternative, coherent, adaptive strategies: proactive individuals tend to dominate and outcompete reactive ones in a stable environment, while reactive individuals appear to respond better to changing environments. In addition, proactive and reactive individuals have been linked to low and high stress axis outputs, respectively (low and high cortisol or corticosterone concentrations). Therefore, identification of such intra-specific differences in cortisol responsiveness and better understanding of their impact in the animal performance and fitness would be beneficial towards better husbandry and selection of breeding stocks in genetic selection programs.

The European sea bass (*Dicentrarchus labrax* L.) is an economically important marine fish and along with gilthead sea bream (*Sparus aurata*), they are the two leading species of Mediterranean aquaculture in the last four decades; in 2016, 191.003 metric tons (tn) of European sea bass were produced globally with a market value of more than 950 million Euros [9]. With the increase in hatchery production, selective breeding of European sea bass received growing interest, while several studies reported a medium to high estimated heritability of growth [10,11,12], revealing a great potential of the species for genetic improvement of commercially important traits. Till now, there are thirteen breeding programs recorded for European sea bass, the majority of which perform family selection on growth performance. The reported number of selected generations varies between two and eight with a cumulative genetic gain in harvest weight estimated to range from +50 to +150%. According to Vandeputte et al. [12], selective breeding for growth in European sea bass has led to 23% increase in harvest weight in four generations of selection.

It is generally accepted that stress causes decreased immune function in fish and affect reproduction by altering levels and patterns of reproductive hormones that influence maturation. The European sea bass is a species with high susceptibility to stress, displaying high basal (resting) cortisol concentrations, as well as intense cortisol response following exposure to acute stressors [13]. There are relatively few published data on the genetic variation of the hormonal cortisol stress response in this species, with a heritability estimate of 0.08 ± 0.06 [14] and three suggestive QTL [15]. Samaras et al. [16] provided strong evidence that post-stress plasma cortisol concentration is a repeatable trait in sea bass individuals (r = 0.39), and it was possible to distinguish between fish that showed a consistently high or low acute stress cortisol response (HR and LR, respectively). There were also significant differences in the basal cortisol concentrations, with HR showing higher levels of total and free circulating cortisol levels than LR individuals, accompanied by higher interregnal sensitivity to adrenocorticotropic hormone (ACTH) signaling [17]. Moreover, a moderate heritability (h^2^ = 0.34 ± 0.09) for mean post-stress cortisol levels and a moderately negative correlation with body weight (r_A_ = −0.36 ± 0.18) was reported in a recent study [18]. Therefore, sensitivity to stress might be an important selection criterion in a sea bass breeding program, as selection for growth could possibly lead to negative correlated responses of the selected fish regarding this trait.

Infectious diseases are, together with feed consumption, probably the sector where more fish producers report highest expenses. Hence, it is of main importance to apply sustainable strategies to reduce the use of therapeutics in the production systems. Genetic improvement in other fish species has shown that well planned family-based breeding programs yield to genetic gains on disease resistance-related traits greater than 12% per generation when relevant challenge tests are applied [19]. A promising protocol to challenge European sea bass against vibriosis has been developed, and mortality due to the pathogen varied from 52% to 57% of the fish challenged; data analysis showed a moderate-high heritability value of 0.15 ± 0.04 for survival on observed scale [20]. In the case of pasteurellosis, a challenge test has also been developed and validated, in which approximately 55% of the fish exposed to the pathogen were affected and died due to the disease; using the data collected from this test, a heritability value of 0.27 ± 0.06 for survival was estimated [20]. Results from both tests are promising and estimated heritability shows great potential for implementation in breeding programs.

In this context, 25% of the genome scan was performed using microsatellite markers to locate possible QTL affecting body weight, three stress and immunological indicators (cortisol, glucose, and lysozyme levels) and mortality after vibriosis injection. Furthermore, heritability and genetic/phenotypic correlations were estimated for all traits under study. The main objectives of this study were to provide genetic tools that are necessary to improve more efficiently important traits in European sea bass breeding, such as growth, stress response, and disease resistance, as well as, to get a better insight into the correlation profile of all these traits. Such results could give us the additional knowledge needed in order to advance the species’ breeding program to the next level.

## 2. Materials and Methods

### 2.1. Ethical Statement

All experiments were performed in accordance with relevant guidelines and regulations. Nireus S.A. research facilities are certified and have obtained the codes for the rearing and use of fish for scientific purposes (EL04-BIOexp-01). All procedures on fish used in this study were approved by the Departmental Animal Care Committee following the Three Rs principle, in accordance with Greek (PD 56/2013) and EU (Directive 63/2010) legislation on the care and use of experimental animals.

### 2.2. Study Design

Stress tests for a three-month period, once per month, were performed on 960 sea bass fish that had been hatched from February 2014 to March 2014, as previously described by Pottinger and Carrick [21,22] and modified by Fanouraki et al. [13] and Samaras et al. [23]. Briefly, fish were exposed to chasing stress for 5 min and confinement to 1/3 of the initial volume of the tank for 30 min. After that fish were immediately anesthetized in ethylene glycol monophenyl ether (300 ppm; Merck; 807291; USA) and blood sampled. Blood was collected from the caudal vessel via heparinized syringes and centrifuged (2000 g; 10 min), and the resulting plasma was stored at −20 °C until analyzed. The 960 fish originated from individual artificial mating between 90 male and 33 female European sea bass brooders, constituting 96 full-sib families (10 fish per family). Fish from each family were placed in one 140 L circular tank, provided with filtered and UV treated seawater (30 ppt) at the rate of 1 L exchange in every hour. Lighting was 12 h light and 12 h dark; water temperature fluctuated between 17–19 °C and dissolved oxygen 6–12.2 ppm. Since the fish were part of a breeding program of a commercial company, pedigree records were available for all specimens which were also PIT (Passive Integrated Transponder) -Tagged.

Seven months after stress test experiment, in order to explore the mortality after *Vibrio anguillarum* injection, a part of the 960 fish [637 offspring with a mean body weight at 115 g (ranged from 20 to 308 g)] were randomly selected to represent each family (4–7 offspring per family) to get involved in a pilot study. Seven different concentrations of *Vibrio anguillarum* serotype I, isolated from reared E. sea bass in Western Greece, were tested and the optimal dose (3.36 × 103 cfu mL^−1^) of the bacterium was determined, based on mortality rates in the following days. In the disease resistance, experiment fish were placed in one 8 m^3^ circular tank (all fish were placed in the same tank), provided with filtered and UV treated seawater (38.5–39.0 ppt) at the rate of 1 L exchange in every 5 h. Lighting was in natural circle; water temperature fluctuated between 19–23 °C and dissolved oxygen 6.7–12.7 ppm. Fish infested with an injectable dose of 0.1 mL of 3.36 × 103 cfu mL^−1^ *Vibrio anguillarum* and mortality started on the second day after infestation. Dead fish every 4 h were removed and recorded.

### 2.3. Measurements

In the stress test, the body weight was measured and blood samples were collected on days 290–306 (bwt1), 318-334 (bwt2), 346–362 (bwt3), and 362–378 (bwt4) after hatching. The range in “days post-hatch” (DPH) is due to the required length (16 days) that needed to produce the 96 families. Plasma concentration of biochemical markers (glucose and lysozyme levels) and hormone markers (cortisol level) was measured. Plasma cortisol level was assayed by a commercial ELISA kit (DRG Control ELISA, DRG International Inc, Springfield, NJ, USA) previously evaluated in European sea bass [23], whereas glucose level was estimated by an enzymatic colorimetric test kit (GOD/PAP) (Biosis, Athens, Greece). Lysozyme was quantified using the turbidimetric assay with *Micrococcus lysodeictikus* (M3770, Sigma-Aldrich, St. Louis, MO, USA) as substrate as described by Lygren et al. [24]. Lysozyme activity was expressed as kUl^-1^ based on a lysozyme (L6876, Sigma-Aldrich, St. Louis, MO, USA) standard curve.

### 2.4. Selective Genotyping

A total of 620 offspring from 62 full sib families (10 offspring per family) out of the initial 96 families were selected for genotyping, based on the within-family variance of cortisol blood level and of body weight. Families with the highest within-family variation were selected; in particular, 24 families were chosen because they were in the 50% of the families with the highest variation in both cortisol level and body weight, 20 families were selected because they were in the top 50% of the families with the highest variation only in body weight, and 18 families were chosen because they were in the top 50% of the families with the highest variation only in cortisol level (File S1).

### 2.5. DNA Extraction and Genotyping

Genomic DNA was isolated from fins preserved in 100% ethanol, using the DNA easy Blood & Tissue Kit (Qiagen, Hilden, Germany). The quality and quantity of DNA were verified using a NanoDrop 1000 Spectrophotometer (Thermo Scientific, Walsham, MA, USA). The DNA concentration of each sample was adjusted to 20 ng/uL through dilution with distilled water and arrayed into 96-well PCR plates. A total of 620 offspring and 110 parents were genotyped for 35 microsatellite markers, distributed in six linkage groups (LG1, LG3, LG4, LG6, LG14, and LG23).

Linkage groups analyzed in this study were selected based on previous reported QTL on stress response and body weight [10,15,25]. According to the first- and second-generation linkage maps of the species, the total distance of map (306.1cM) [26,27], the distribution on the linkage groups, and polymorphism of the available microsatellite markers, 35 markers were selected in order to secure sufficient power of QTL [28,29].

Multiplex PCR reaction method, amplifying simultaneously the above microsatellite loci, was carried out (Table S1). All multiplex PCRs were performed in 10 μL volume containing 6 μL of 1× KAPA2G Fast Multiplex PCR Kit (KAPA BIOSYSTEMS, Wilmington, MA, USA), 3 μL of primer mix (0.3 μM for each primer), and 1 μL (~20 ng) of template DNA. Cycling conditions for the multiplex amplification consisted of an initial 95 °C denaturation step for 3 min followed by 35 cycles of 30 sec at 94 °C, 90 sec at 60 °C, and 60 sec at 72 °C, with a final extension at 72 °C for 10 min. Fluorescently labeled PCR products were separated on an ABI 3730 DNA Analyzer (Applied Biosystems, Foster City, CA, USA). Alleles were sized and individuals genotyped using the STRand 2.4.59 software [30].

### 2.6. Statistical Analysis

For the following variables: cortisol, glucose, lysozyme levels, body weight, and mortality from *Vibrio anguillarum* injection, genetic parameters, including heritability, genetic correlation, and phenotypic variation, were estimated using REML methodology with WOMBAT 1.0 software (University of New England, Armidale, Australia) through an animal model (repeatability model with no fixed effects present). Although weight was fitting as covariate, it did not affect the estimation of genetic parameters. Pedigree records were utilized during the aforementioned analysis. Survival analysis was performed in SPSS 21.0 (IBM, New York, USA) in order to quantify mortality after *Vibrio anguillarum* injection. Specifically, the Survival Function and Hazard Function were used to estimate survivability and the instantaneous probability of death occurring at the end of the experiment (hazard ratio), respectively. The survivability and hazard ratio as well as the binary values (0/1, dead/alive) from the Vibrio experiment were used to estimate genetic parameters for the disease resistance trait and for the detection of putative QTL. Only 406 (out of the total 637 offspring participating in vibriosis experiment) were used for the genetic parameters estimation since only for those fish complete records for all traits studied (cortisol, glucose, lysozyme levels, body weight, and mortality after *Vibrio anguillarum* injection) were available.

A de novo linkage analysis was performed in order to identify the linkage, the position, and the distance between the 35 microsatellite markers distributed in six linkage groups, using CRI-MAP 3.0 statistical package [31]. Briefly, assignment of the markers to LGs was performed by pairwise analysis (‘two-point’ option), and LGs were built assuming an equal recombination rate between sexes. The linkage distances for sex-average and sex-specific LGs were estimated assuming the Kosambi’s mapping function. QTL analysis for the aforementioned traits and the genotyped microsatellites was undertaken using a maximum likelihood Variance Component Analysis with: (a) one QTL model and (b) a mixed model (polygenic and QTL effects) with the presence of a polygenic factor (pedigree-based animal model). In the first case, the analysis indicated putative QTL positions and provided estimated effects and in the second case, the polygenic component was included in the analysis of the putative QTL in order to take into account spurious genetic effects (QTL and polygenic effects) from the rest of the genome that was not under investigation and thus obtain more unbiased QTL effects. The Qxpak 5.0 software [32] was used for the analysis in all cases.

## 3. Results

The descriptive statistics of the measured traits under study are presented in Table 1. There were three repeated measurements on stress and metabolic and immunological markers (cortisol, glucose, and lysozyme levels) after repeated stress test [16].

### 3.1. Estimation of Genetic Parameters

Heritability of cortisol, glucose, and lysozyme levels was medium to high (0.37, 0.23, and 0.56, respectively) (Table 2). Genetic correlation between mortality (as binary 0/1 trait) and cortisol level was medium to low (0.33) (Table 3). Moreover, a negative genetic correlation (−0.43) was found between cortisol level and body weight. However, the phenotypic correlations between weight and cortisol, glucose, or lysozyme levels were almost zero (Table 2).

Genetic and phenotypic correlations between biochemical (glucose and lysozyme levels) and hormonal markers (cortisol level), as measured after the end of the stress test and before the challenge test with *Vibrio anguillarum* (which took place 7 months later), with mortality during the challenge test were low (0.33) and in some cases negative (e.g., −0.18). However, the heritability of mortality was quite high (0.38) given the nature of the trait (Table 3).

Here was no significant genetic correlation between weight and mortality from *Vibrio anguillarum* or its hazard function (Table 4). Repeatability between the three measurements of cortisol, glucose, and lysozyme levels were 0.46, 0.42, and 0.61, respectively.

### 3.2. Genetic Map

**Figure 1:**
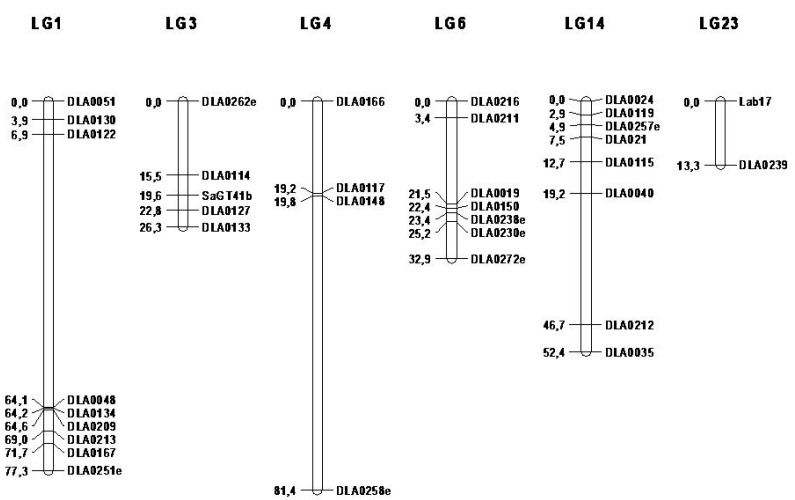
Sex-averaged genetic map comprising of 35 markers in six linkage groups. Linkage groups are named according to the first- and second-generation linkage maps of sea bass [26,27].

A partial genetic linkage map was constructed de novo with a sex-averaged length of 283.6 cM and a mean marker interval (across the six LGs) of 8.1 cM, which is of satisfactory marker density according to the required interval of 10–20 cM for sufficient power of QTL mapping [28,29]. The linkage map is, generally, in good agreement with previous genetic linkage maps [10,15,25,26,27], although in most of the groups, order and distance of markers are not totally preserved. Such differences are expected between studies due to the use of different families (and therefore, populations of different genetic backgrounds). The complete set of LGs with orientation and distance of markers is presented in Figure 1.

### 3.3. QTL

**Figure 2:**
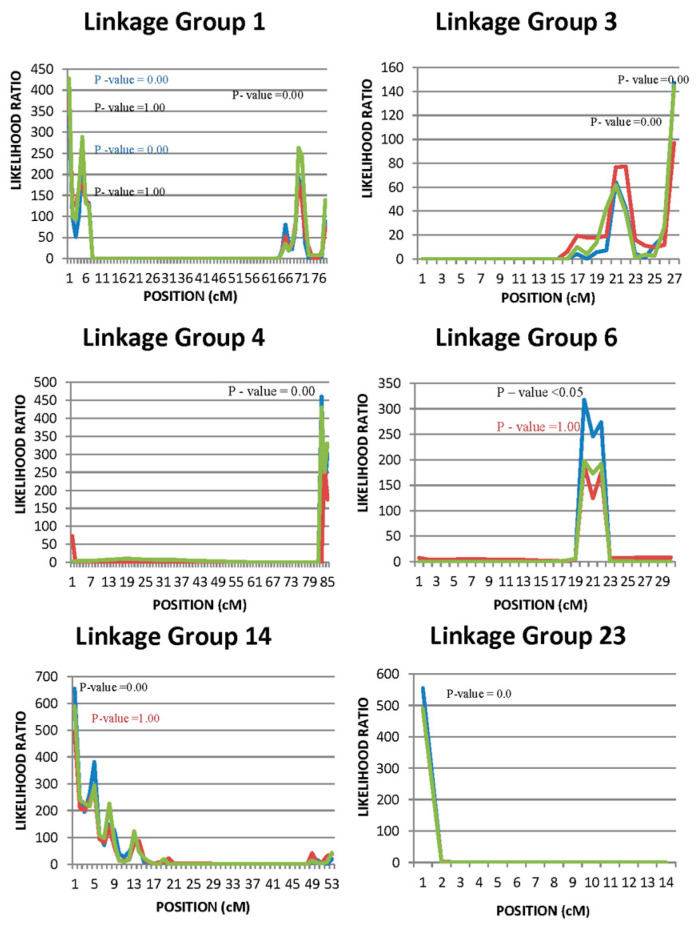
Quantitative trait loci (QTL) analysis (using a mixed model which includes a polygenic component) of cortisol, glucose, and lysozyme in different linkage groups. Blue, red, and green lines represent likelihood ratios of cortisol, glucose, and lysozyme levels, respectively. Blue, red, and green *p*-values represent significance of cortisol, glucose, and lysozyme levels, respectively. Black color represents significance of all traits.

QTL analysis confirmed the existence of QTL affecting cortisol level in linkage groups 3 and 14. The same QTL seem to have a pleiotropic effect on other stress, metabolic, and immunological indicators as well, i.e., the blood biochemical markers glucose and lysozyme levels. On the other hand, no significant linkage was found between a QTL located in linkage group 23 and cortisol level, however, a QTL associated with glucose level was identified in this position. In addition, a new QTL affecting only cortisol level was detected in linkage group 1. Finally, no QTL affecting hormonal or biochemical markers was found in either linkage groups 4 or 6 (Figure 2). Regarding mortality after *Vibrio anguillarum* injection, no QTL was detected in any of the linkage groups under investigation, when resistance was measured as mortality or survivability (survival function) or hazard function after the vibriosis challenge experiment (data not shown).

**Figure 3:**
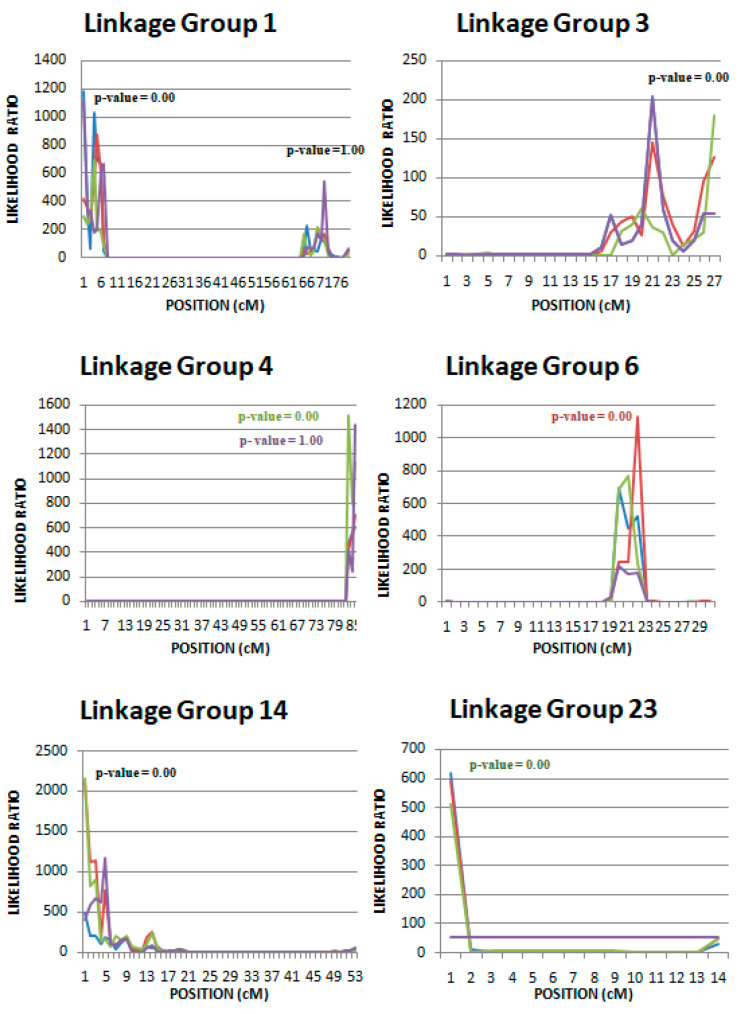
QTL analysis (using a mixed model which includes a polygenic component) of body weight at 290-306 DPH (bwt1), 318-334 DPH (bwt2), 346-362 DPH (bwt3), and 362-378 DPH (bwt4) in different linkage groups. Blue, red, green, and purple lines represent likelihood ratios of bwt1, bwt2, bwt3, and bwt4, respectively. Blue, red, green, and purple *p*-values represent significance of bwt1, bwt2, bwt3, and bwt4, respectively. Black color represents significance of all body weights.

QTL in linkage groups 1, 4, 6, and 14 affecting body weight in three different growth periods [(bwt1 (290–306 DPH), bwt2 (318–334 DPH), bwt3 (346–362 DPH)], were detected. A QTL associated with weight in early growth stages (bwt1) and possibly late growth stages (bwt4) was also identified in LG3. Nevertheless, significant QTL for body weight during the whole growth period studied (290–378 DPH) were found only in linkage groups 1 and 14 (Figure 3). It is worth noting that QTL linked to hormonal (cortisol) and/or biochemical markers (glucose and lysozyme levels) are located in the same regions of LG1 and LG14 as with the body weight QTL (Figure 2 and Figure 3).

The additive values of all detected QTL, as a percentage of the phenotypic variation explained, are summarized in Table 5 and Table 6. Although the estimate additive QTL effects were quite substantial for hormonal and biochemical stress indicator traits in some cases (LG3), they were low in other cases (LG1) and very low or unable to estimate and non-significant in other cases (i.e., lysozyme levels in all LG under the one QTL model (case a)) (Table 5). As far as the body weight estimated, QTL additive effects were low to very low in all linkage groups. Moreover, in some linkage groups (e.g., LG4 in all growth stages) the additive QTL effects was unable to be estimated or the estimates were non-significant. However, the same linkage groups produced medium additive QTL effects in all growth stages under a mixed QTL model (QTL + polygenic, case b) (Table 6). Nevertheless, among the identified QTL, the one with the most notable pleiotropic effect was found close to microsatellite marker SaGT41b in linkage group 3. However, when a mixed model (polygenic and QTL) was used in the analysis, the estimated effects of the QTL were minimized (Table 5 and Table 6).

## 4. Discussion

The heritability of body weight (bwt4) was estimated in a multitrait animal model as 0.38, but when the body weight was measured 7 months after the stress test experiment (520 DPH) and used in a multitrait animal model, together with the biochemical and hormonal markers measurements, its heritability estimate increased (0.61, data not shown) and it was of a similar estimate with the multitrait analysis for mortality in the vibriosis challenge test (0.38, Table 3). Analyzing only biochemical and hormonal markers in a multitrait animal model, their heritability estimates remain unchanged, but the genetic and phenotypic correlation between glucose, lysozyme, and cortisol levels decrease. More specifically, the most significant drop was the genetic correlation between cortisol and glucose levels (about a third of its initial value) (data not shown). In the multitrait animal model that included mortality after *Vibrio anguillarum* injection, the heritability of glucose decreases by 1/3 (0.23 from 0.33 (multitrait animal model included bwt4)) and the rest of the heritability estimates (Cortisol, Lysozyme levels) remain constant (Table 2 and Table 3).

It seems that growth impairment following exposure to repeated stress masks juveniles’ body weight heritability estimates in a multitrait animal model, with the exception of glucose level needed to cover the higher energy demands to restore homeostasis. However, in subsequent stages of development (immature 7-months old fish), a possibly non-additive genetic relation between cortisol, glucose levels, and body weight results in decreased glucose levels and increased body weight heritability estimates.

Volckaert et al. [14] estimated a very low heritability (0.08) of blood cortisol level under stress conditions. However, they did not use a repetitive stress experiment like the present study, and the stressor technique applied was mild (decrease of the water level). From all biochemical and hormonal markers, only cortisol and lysozyme levels had a steadily high heritability estimate, irrespective of the trait included in the model (0.36–0.37 and 0.55–0.56 (Table 2 and Table 3)). A combination of those results and the similarity of the estimations of heritability and repeatability for those traits (0.24 and 0.54, respectively (in a multitrait analysis of this study)), and Samaras et al. (univariate estimates) [16] can give an initial indication of a major gene segregation within the population under study.

Furthermore, the biochemical (glucose), immunological (lysozyme), and hormonal (cortisol) markers under study did not have significant phenotypic correlation with the weight of the fish recorded during the blood sampling of the stress test experiment. However, there was a negative genetic correlation (−0.43) between cortisol level and body weight of the fish (Table 2). These results could mean that the better the genetic potential for growth of a fish, the lower the cortisol level that it is producing. Or, in other words, fish that produce less cortisol level when stressed have better growth potential.

In addition, there was a change in the heritability estimates of blood glucose levels depending on the trait included in the multitrait animal model (body weight (0.23) or mortality (0.33) (Table 2 and Table 3)). These differences in the heritability estimates could be due to the negative correlation of body weight with cortisol level in the blood and the positive correlation with the glucose level and it could be an initial indication of possible pleiotropy and/or epistasis. Nevertheless, the existence, not only of genetic correlation between glucose and cortisol levels but also the significant change of the heritability of glucose level with different analysis models, requires further research.

Moreover, mortality exhibits an unusual high heritability (0.32). The aforementioned estimate might be affected by the challenge testing nature of the experiment (direct contamination of the fish) in order to provoke the outbreak of the disease. Consequently, the frequency of dead fish was increased. However, when survival analysis data were used there was significant differentiation in the heritability estimates of mortality-related traits (i.e., survivability, Hazard Function) (Table 3 and Table 4). Nevertheless, it was not possible to distinguish if this differentiation in the estimates was a result of the nature of the experiment (invasive direct contamination) and if these heritability estimates would be valid in commercial farmed populations under normal conditions of the disease outbreak when the virus is found in the water. Moreover, the disease resistance, caused by *Vibrio anguillarum*, does not seem to be significantly correlated with any of the biochemical (glucose and lysozyme levels) or hormonal (cortisol levels) markers used in this study or by the body weight of the fish. In addition, when phenotypic correlations were calculated as family’s average, only the correlation with mortality was statistically significant but low (0.113, *p* < 0.05). It is notable that, even though heritability of mortality is higher than other diseases, no QTL linked with mortality, survivability, or hazard function was found during the present analysis. Further research should be done because only 25% of the genome was explored. Furthermore, evidence of a major SNP (Single Nucleotide Polymorphism) effect on VNN (Viral Nervous Necrosis) has been reported on a genetically related population of sea bass [33]. Since major gene segregation for disease resistance in European sea bass is a plausible scenario, high heritability estimates of mortality of Vibrio may also be possible. Genome-wide association analysis for Vibrio is in progress and the results could explain the high genetic variance component of the experiment herein.

The findings of the present study regarding the existence of QTL, which affect plasma levels of cortisol levels in linkage groups 3 and 14, are consistent with those of Massault et al. [15] in a broodstock of different origin. However, in our study, no linkage between a QTL located in linkage group 23 and cortisol levels, as reported by Massault et al. [15], was identified. Furthermore, new QTL affecting glucose level have been detected in the aforementioned linkage groups, as well as new QTL affecting cortisol level in linkage group 1 and also glucose in linkage group 23. The identification of QTL in the same linkage groups (LG1 and LG14) affecting cortisol, glucose, lysozyme levels, and also body weight reinforces the argument for possible pleiotropic QTL effects.

The change of the estimated additive effects of QTL under different analysis models (single QTL genetic model vs. mixed model (polygenic + QTL)) (Table 5) could suggest three different scenarios: (a) the QTL might not be located in the genetic regions assessed in the present study, (b) the inclusion of a polygenic component in the analysis and the pedigree relationship matrix values might follow the genetic relationship matrix based on the marker genotypes, thus creating an inflating effect on the test statistic and the parameter estimates (i.e., QTL effects), and (c) the QTL effects might not be just additive, but they might be pleiotropic or even epistatic. Thus, further investigation of the interactions (epistatic effects) among the identified QTL, as well as their fine-mapping is required prior to their usage into breeding programs.

With regard to weight, QTL were identified in linkage groups 1, 3, 4, 6, and 14 under both one QTL and/or mixed model. However, in linkage groups 3, 4, and 6, statistically significant linkage was identified only in the early stages of growth (bwt1, 209-306 days after hatching). Recently, Louro et al. [25] reported statistically significant QTL for growth in linkage groups 4, 6, and 15 (Linkage group 15 was not explored as part of the present study). Weight and morphometric traits (such as length) of fish analyzed in the study by Louro et al. [25] correspond to age of 240 days after hatching, thus in the same age as, in the current study, QTL were identified in the same linkage groups (LG4 and LG6). Two QTL were reported affecting body weight in linkage groups 4 and 6 (Massault et al. [15]) (average body weight was equal to 40.58 gr). Moreover, Chatziplis et al. [10] reported a potential QTL affecting body weight (average body weight 463.5 g) on LG1. Further study of the interactions among QTL (epistasis) might illuminate the expression of these QTL in different stages of growth. A list of genes located in the surrounding regions of the detected QTLs is also presented in Table S2 according to the European sea bass genome database (http://seabass.mpipz.mpg.de) [34].

Nevertheless, the high heritability estimated for cortisol and lysozyme blood levels (Table 2) together with the detected QTL (Figure 2 and Figure 3) are strongly suggesting the segregation of a major gene(s) affecting these traits within the population under study. A more detailed genetic research including a large number of genetic markers (i.e., SNP markers through NGS (Next Generation Sequencing) techniques) could provide more information in terms of fine mapping any putative QTL and/or identify possible alternative modes of inheritance (i.e., epistasis) and presence of pleiotropy. The experiments of this study will be further investigated and fish from both experiments (old and new) will be genotyped with thousands of SNP markers in order to fine map detected QTL and identify any new ones. Such information could be very useful in terms of incorporation of any positive results into a breeding program via Marker-Assisted Selection or even Genomic Selection in the case of more extensive genomic data availability.

## 5. Conclusions

Investigating the growth, stress response, and disease resistance and their relationships (both genetic and phenotypic) in European sea bass could improve the species’ current breeding programs. Studying those traits improved the knowledge of inheritance and their correlations. Moreover, QTL affecting cortisol levels were confirmed (LG3 and LG14) and seem to have pleiotropic effects on other stress indicators. Furthermore, new QTL in LG23 and in LG1 are detected to affect glucose and cortisol levels, respectively. Nevertheless, body weight in different stages was affected by QTLs in LG1, LG3, LG4, LG6, and LG14. Finally, no QTL affecting the resistance against *Vibrio anguillarum* were found.

## Figures and Tables

**Table 1:** Total number of measurements, mean, standard deviation, and range of values per trait.

Traits	N (Total Number of Measurements)	Mean ± SD ^1^	Range	
			Min	Max
Cortisol level (ngmL^−1^) *	1860	324.4 ± 86.0	31.7	547.4
Glucose level (mmolL^−1^) *	1833	7.42 ± 2.26	1.1	24.5
Lysozyme level (kUL^−1^) *	1720	677 ± 326	68.0	5065.0
bwt1 (g)	616	38.9 ± 10.6	14.0	90.0
bwt2 (g)	618	43.9 ± 13.1	13.9	114.2
bwt3 (g)	614	52.5 ± 16.0	13.6	142.3
bwt4 (g)	600	56.4 ± 17.9	12.3	143.5

* Repeated measurement (3 measurements per fish). ^1^ SD = Standard Deviation.

**Table 2:** Estimates of heritability (bold), genetic correlation, and phenotypic correlation (above and below the diagonal, respectively). Standard errors are in parenthesis.

	Cortisol Level	Glucose Level	Lysozyme Level	Bwt4
Cortisol level	**0.37 (0.08)**	−0.24 (0.20)	−0.14 (0.14)	−0.43 (0.22)
Glucose level	−0.04 (0.04)	**0.23 (0.09)**	0.04 (0.15)	0.08 (0.26)
Lysozyme level	−0.03 (0.04)	−0.01 (0.04)	**0.56 (0.07)**	0.05 (0.16)
bwt4	0.08 (0.05)	0.02 (0.05)	0.04 (0.04)	**0.35 (0.13)**

**Table 3:** Estimates of heritability (bold), genetic correlation, and phenotypic correlation (above and below the diagonal, respectively). Standard errors are in parenthesis.

	Cortisol Level	Glucose Level	Lysozyme Level	Mortality after *Vibrio anguillarum* Injection
Cortisol level	**0.36 (0.08)**	−0.25 (0.19)	−0.14 (0.14)	0.33 (0.26)
Glucose level	−0.03 (0.03)	**0.33 (0.06)**	0.04 (0.14)	0.11 (0.28)
Lysozyme level	−0.03 (0.03)	−0.01 (0.03)	**0.55 (0.07)**	−0.18 (0.19)
Mortality after *Vibrio anguillarum* injection	0.12 (0.05)	0.05 (0.05)	−0.07 (0.05)	**0.38 (0.18)**

**Table 4:** Estimates of heritability (bold), genetic correlation, and phenotypic correlation (above and below the diagonal, respectively).

	Mortality after *Vibrio anguillarum* Injection	Survivability	Hazard Function	Body Weight ^Ɨ^
Mortality after *Vibrio anguillarum* injection	**0.32 (0.07)**	−0.92 (0.03)	0.98 (0.01)	−0.11 (0.21)
Survivability	0.32 (0.08)	**0.38 (0.08)**	−0.94 (0.02)	0.16 (0.25)
Hazard Function	−0.30 (0.07)	−0.95 (0.01)	**0.32 (0.08)**	0.12 (0.27)
Body Weight	0.11 (0.08)	0.04 (0.11)	0.00 (0.09)	**0.49 (0.16)**

^Ɨ^ The bodyweight is as measured before the experiment for resistance against *Vibrio anguillarum* (on average 520 DPH) and the measurements took place 7 months after the stress test experiment (therefore, heritability estimates for body weight on Table 1 and Table 4 are estimates for different growth periods).

**Table 5:** Phenotypic variation of all QTLs (QTL heritability) for stress indicator traits: cortisol, lysozyme, and glucose levels.

	QTL Expressed as % of the Phenotypic Variation		MIXED MODEL (QTL + POLYGENIC) Expressed as % of the Phenotypic Variation					
	LG1	LG3	LG14	LG23	LG1	LG3	LG14	LG23
Cortisol level	2.84 *	31.35 *	0.17 *	0.20 *	0.32 *	2.52 *	0.15 *	0.07 *
Glucose level	5.02 *	7.80 *	0.49 *	0.34 *	0.74 *	2.94 *	0.28 *	0.17 *
Lysozyme level	-	-	-	-	0.19 *	4.59 *	0.96 *	0.43 *

* *p*-value < 0.05.

**Table 6:** Phenotypic variation of all QTLs (QTL heritability) for bodyweight.

	QTL Expressed as % of the Phenotypic Variation					MIXED MODEL (QTL + POLYGENIC) Expressed as % of the Phenotypic Variation				
	LG1	LG3	LG4	LG6	LG14	LG1	LG3	LG4	LG6	LG14
bwt1	2.38 *	1.12 *	-	0.70 *	1.02 *	<0.01 *	0.12 *	6.06 *	<0.01 *	<0.01 *
bwt2	1.27 *	4.45 *	-	1.04 *	0.70 *	<0.01 *	0.01 *	6.28 *	<0.01 *	<0.01 *
bwt3	1.34 *	5.14 *	-	2.63 *	0.99 *	<0.01 *	4.78 *	5.64 *	-	10.00 *
bwt4	3.44 *	7.90 *	-	-	2.05 *	<0.01 *	0.01 *	4.57 *	4.92 *	<0.01 *

* *p*-value < 0.05.

## Supplementary Materials

The following are available online at https://www.mdpi.com/2076-2615/10/9/1668/s1, File S1: Statistical parameters of selected sea bass families, Table S1: Information for used microsatellite markers, Table S2: List of genes located in the surrounding regions of the detected QTLs per linkage group.
