# Supplementary material for: QTL for Stress and Disease Resistance in European Sea Bass, Dicentrarhus labrax L
Source: Animals (Basel). 2020 Sep 16;10(9):1668. doi: 10.3390/ani10091668 (PMC7552151; doi:10.3390/ani10091668)
Supplement: Supplementary file 1 [file animals-10-01668-s001.zip › Supplementary Materials/Table S1.docx]

| **Markers** | **Forward Primer** | **Reverse Primer** | **Multiplex Number** | **Fluorescent dye** | **Allelic range (bp)** | **Linkage Group** |
| --- | --- | --- | --- | --- | --- | --- |
| *DLA0262e* | GGAACGTGTAAATTACCATCCTCA | ACCCACGCTCAAGCTAACTC | 1 | FAM | 100-140 | 3 |
| *DLA0133* | TTAGCATATTAGCACGCTATC | ACTCTCACTTCAGGCACAAG | 1 | FAM | 172-198 | 3 |
| *DLA0114* | CACTGACCCATGGCCTAACT | GAGTCCTTGCGCGTGAATA | 1 | ROX | 148–206 | 3 |
| *SaGT41b* | GGAGGCACTTGCAAGTGCAG | AGATGGCAGAGGTCTGGTAG | 1 | TAMRA | 150-202 | 3 |
| *DLA0239* | CAGCTCCGGAGGTCTGTAAG | GGCCTGTTGGTGGCATTAT | 1 | HEX | 193-249 | 23 |
| *DLA0212* | CCCAGTTTTAGCCCTCAGTC | TGGTGGTTTTGTCAGCACTC | 2 | ROX | 202-226 | 14 |
| *DLA0035* | ACAAGAGTGTGTAACTACGTG | AGGGAAGAACTATAACGATG | 2 | HEX | 187-193 | 14 |
| *DLA0257e* | CTGGGAGAACCAGCCTACAA | AGGAGGCCAAATCAACCTTT | 2 | HEX | 134-155 | 14 |
| *Lab17* | TGTCTTCTGCCAACTCAC | TGAAGGCAAGAGGGAAAC | 2 | FAM | 117-163 | 23 |
| *DLA0127* | ACTGGATGGCACACACTGAA | CAGAAATGAGGTTACATTGCTGAG | 3 | ROX | 153-169 | 3 |
| *DLA0115* | GCAGGATATGAGATAAGTGCCTAAA | TGAGAGCTCACTGGAAGCTG | 3 | HEX | 139–165 | 14 |
| *DLA0040* | CCCCTAACGTAGTTTTATCTCCA | GCTGCTGAACACCATAACAA | 3 | FAM | 270-290 | 14 |
| *DLA0119* | GCAGGTTCAAATTATTTTTGCTC | TCCTCCTTTTGCTTGCTAGG | 3 | HEX | 219–261 | 14 |
| *DLA0024* | TCAAAACACTGCCAGCTGAA | AAAAGCATTTCTCGTCTGCAC | 3 | FAM | 98-132 | 14 |
| *DLA0051* | AGGTTCTTGGCCTGGGAATC | AGTGACAGCAGCCTCCAGAG | 4 | FAM | 149-181 | 1 |
| *DLA0213* | GTCGATCGAATGACCTGTTGT | ATGCACACCATCAGGCATAA | 4 | FAM | 100-112 | 1 |
| *DLA0122* | GGCTTCTTATGTGTTGGACT | GTAAATTTGCATACAGAAGCA | 4 | HEX | 226-244 | 1 |
| *DLA0209* | TGTGTCTGTGCTTTGGGAAG | CCCTTTCCATTCTTGCAAAC | 4 | HEX | 184-198 | 1 |
| *DLA0134* | TCCTCCACACTCTGCACATC | ACATTTTCCTCATCCTTTTTCTT | 4 | TAMRA | 130-166 | 1 |
| *DLA0021* | AGCTCCAATCAGGCAGACAC | GACGGGCACACACATGATTA | 4 | HEX | 121-153 | 14 |
| *DLA0130* | TCTGTGGCAGAATGTGTCTGA | AACCATCACAGCTTGAGTTCC | 5 | FAM | 163-177 | 1 |
| *DLA0167* | ATTTCTCCGCAAAGCACTGA | GCCAGTTCAAGGGCATTACT | 5 | FAM | 214-234 | 1 |
| *DLA0251e* | TGTGATTGATGTCCCAGACTT | GGCAGAATTAAGACTCCGATAA | 5 | HEX | 137-165 | 1 |
| *DLA0117* | TTTGGTAGGTTGCTGAAATGG | GGCTGATCGGTGTATGTCAA | 5 | HEX | 214–240 | 4 |
| *DLA0148* | CACTTGGTAAAGGACGGAACA | AAGGAGGAATCACTGCGTTT | 5 | ROX | 149-167 | 4 |
| *DLA0019* | CCTATTATCTGATGTGTTTATGC | AGTTTCTCCTCACTCGACTCTC | 5 | FAM | 105-139 | 6 |
| *DLA0048* | TGCAGGCTGAATGTGCAGTC | AGGCGCAGAAGAGAGCAGTC | 6 | ROX | 134-156 | 1 |
| *DLA0166* | CAAAAAGCCCGTTGGTTCTA | TGGATCCTGCTGTGAGTGAG | 6 | HEX | 146-156 | 4 |
| *DLA0258e* | CAGTTCTCAAGTTCAACTCTCAA | TCCACCAATCAGAACCTG | 6 | HEX | 183-387 | 4 |
| *DLA0150* | CTCCTCCAGTGCTTGTAGCC | ACAGCTGTTGATGCCCATTT | 7 | FAM | 221-253 | 6 |
| *DLA0230e* | ACCACCCCCTTTATGAGCTT | TGTTCGTGGGACTTCTGTGA | 7 | FAM | 125-161 | 6 |
| *DLA0216* | GAACTCAAACAACAAGGCAAAG | ACCCAGAGTGGAACCATGAA | 7 | HEX | 203-241 | 6 |
| *DLA0272e* | CGTAAGCTCCCTCGCTTT | CTTTCACCTCAAGATATGAAGATCC | 7 | HEX | 141-162 | 6 |
| *DLA0238e* | ACACACTGGGATGTGCAAAA | CACATCCCCTTCACTGAGACT | 7 | ROX | 169-181 | 6 |
| *DLA0211* | CCAAGGCATGTGTCTGAATG | GAGTCTTCCTCCCACACTCG | 7 | TAMRA | 153-161 | 6 |
