# Supplementary material for: QTL for Stress and Disease Resistance in European Sea Bass, Dicentrarhus labrax L
Source: Animals (Basel). 2020 Sep 16;10(9):1668. doi: 10.3390/ani10091668 (PMC7552151; doi:10.3390/ani10091668)
Supplement: Supplementary file 1 [file animals-10-01668-s001.zip › Supplementary Materials/Table S2.docx]

| Linkage Group | Genes |
| --- | --- |
| 1 | DLA_LG1A_007630/LASS5, DLA_LG1A_007620/GPD1 , DLA_LG1A_004890/uncharacterized protein |
| 3 | DLAgn_00143950/suppressor of hairless protein homolog, DLA_LG3_001060/FGF11/fibrioblast growth factor 11 |
| 4 | DLA_UN_008785_3/AAH90917.1/Dihudrolipoamide branched chain transaculase E2(*Danio rerio*),  DLA_UN_0087901/LRRC39 |
| 6 | DLA_LG6_006550/Ap3B2/adaptor_related protein complex 3beta 2 submit, DLA_UN_017630/ZGC:158 603/cyclic AMP-responsive element-binding protein 3-like protein2(cAMP-resposive element-biding protein3-like), DLA_UN_017627/CAG04134,1/unnamed protein product [*Tetraodon nigroviridis*] |
| 14 | DLA_LG14_001390/JAM3, DLA_LG14_002000/CLTC/clathrin heavy chain1, DLA_LG14_002005/ACQ58142.7/ATP synthase subunit g |
| 23 | DLA_UN_002830/SFMT2/Scm-like with four mbt domain 2,  DLA_UN_002840/RAB21 |
